# Early signs of cancer present in the fine detail of mammograms

**DOI:** 10.1371/journal.pone.0282872

**Published:** 2023-04-05

**Authors:** Emma M. Raat, Karla K. Evans

**Affiliations:** Department of Psychology, University of York, York, United Kingdom; University of Wisconsin-Eau Claire, UNITED STATES

## Abstract

The gist of abnormality can be rapidly extracted by medical experts from global information in medical images, such as mammograms, to identify abnormal mammograms with above-chance accuracy—even before any abnormalities are localizable. The current study evaluated the effect of different high-pass filters on expert radiologists’ performance in detecting the gist of abnormality in mammograms, especially those acquired prior to any visibly actionable lesions. Thirty-four expert radiologists viewed unaltered and high-pass filtered versions of normal and abnormal mammograms. Abnormal mammograms consisted of obvious abnormalities, subtle abnormalities, and currently normal mammograms from women who would go to develop cancer in 2–3 years. Four levels of high-pass filtering were tested (0.5, 1, 1.5, and 2 cycles per degree (cpd) after brightening and contrast normalizing to the unfiltered mammograms. Overall performance for 0.5 and 1.5 did not change compared to unfiltered but was reduced for 1 and 2 cpd. Critically, filtering that eliminated frequencies below 0.5 and 1.5 cpd significantly boosted performance on mammograms acquired years prior appearance of localizable abnormalities. Filtering at 0.5 did not change the radiologist’s decision criteria compared to unfiltered mammograms whereas other filters resulted in more conservative ratings. The findings bring us closer to identifying the characteristics of the gist of the abnormal that affords radiologists detection of the earliest signs of cancer. A 0.5 cpd high-pass filter significantly boosts subtle, global signals of future cancerous abnormalities, potentially providing an image enhancement strategy for rapid assessment of impending cancer risk.

## Introduction

Breast cancer is (one of) the most prevalent and deadly cancers in women world-wide, according to global data from 1990 to 2015 [1] and 2020 GLOBOCAN cancer statistics [2]. As with most cancers, early detection is vital, as it allows for treatment before the disease progresses and improves clinical outcomes [3]. Currently, the most commonly used methods of screening and early detection are clinical breast exams and digital mammography, as they are effective and cost-efficient [3] and have been estimated to reduce mortality by 30% to 50% [4]. Digital mammography is especially for early detection, as it allows detection of small, pre-clinical tumours of <15mm that are not detectable with a clinical breast exam [4]. However, 20–30% of cancers are still estimated to be missed during screening in North America [5,6].

Further reducing breast cancer mortality through screening could be achieved by increasing screening frequency. However, increasing screening frequency across the entire population is not cost-effective, and risks increasing false positives [7] or even over-diagnosis of benign breast conditions, which has been associated with unnecessary cost [8] and negative mental health effects [9,10].

Instead, women at an increased risk should be offered more frequent screening. Currently, at-risk women are often identified through familial history of breast cancer, or genetic markers, such as BRCA1 or BRCA2 mutations, which cause approximately 60% of hereditary breast cancer [11]. However, gene screening is costly and BRCA1 or BRCA2 mutations cause only 5% of breast cancer, limiting applicability to the general population. An alternative, more universal approach would be to identify at-risk women based on perceptual features in their existing mammograms. This method relies on the robust observation that experienced radiologists can capture both current and future cancer risk in the blink of an eye through extraction of the gist of abnormality.

This gist of abnormality is extracted through a process that rapidly and non-selectively extracts global structure and statistical regularities from our visual environment [12,13]. In normal observers, this allows them to categorize a flashed scene (30 ms) as a beach or a forest with high accuracy [14,15]. In addition, medical experts are extract the gist of medical images, allowing them to distinguish normal from abnormal cases after 100 to 500 milliseconds of viewing time for chest radiographs [16], cytological images from PAP smears [17], and mammograms [17,18]. Importantly, mammograms of women taken 3 years prior to their eventual diagnosis (priors), that did not contain detectable cancer even when viewed retrospectively, are scored as significantly more abnormal than mammograms of women that did not go on to develop cancer in the near future [19,20]. Thus, the gist of abnormality is a robust signal that can rapidly be extracted from mammograms.

Thus, a high gist of abnormality score could be a promising risk factor to flag mammograms for a secondary opinion (current risk) or to recommend women for more frequent scanning (future risk). Advantages of the gist signal are that it can be extracted from already existing mammograms, and it is already visible in cases up to 3 years prior to cancer onset, without visibly actionable lesions. Unfortunately, the signal strength in priors is relatively weak with an observed d’ of 0.22 and an Area Under the Curve (AUC) of 0.54–0.6 for priors without visible abnormalities [19,20]. Thus, methods to strengthen the gist of abnormality signal, especially in priors, are needed to make it more clinically viable.

Spatial frequency filtering might allow a way to isolate and enhance the perceptual features that comprise the gist signals in mammograms. Visual information can be summarized as spatial frequencies in various orientations. Low spatial frequencies (LSF) provide coarse information spread across a large area, whereas high spatial frequencies (HSF) provide finer details of for example edges and contours. Together, LSF and HSF provide important information about the texture and structural regularities in our visual environment. But it is possible that the gist of abnormality is stronger in specific frequency bands, or that it is masked by other frequency bands that make it harder to perceive. Interestingly, filtering out HSF strongly reduced accuracy of rating normal vs abnormal mammograms from a d’ of 1.06 with full spectrum mammograms to only 0.26, while filtering out LSF resulted in a relatively high d’ of 0.96 [18]. Thus, gist of abnormality seems to be preferentially contained in HSF, although there was still a small reduction in performance.

Conflicting findings have been reported on the effects of spatial frequency filtering on general gist extraction. Merged spatial frequencies from two scenes were most frequently perceived as the LSF scene with 30 ms view time, but with 150 ms HSF dominated [21], suggesting an early importance for LSF. However, recent evidence points to the importance of HSF for scene gist when taking contrast normalization into account. Natural images contain more LSF than HSF contrast energy, following an inverse power law [22]. This means that HSF-only images have lower overall visibility. After contrast normalization human observers showed equal performance on gist categorization of LSF and HSF scene images [22].

Since Evans, Haygood [18] did not contrast normalize the mammograms, the reduction in performance for HSF compared to full spectrum mammograms might have be caused by a reduction in contrast energy. Additionally, HSF-retaining filters might differentially affect gist signals in different conspicuities. The current study aimed to investigate the effects of five levels of high-pass spatial frequency filtering on the gist of abnormality in mammograms with three different conspicuities when applying contrast normalization. Contrast normalization was combined with a brightness increase to ensure that the higher spatial frequencies were bright enough to be perceived. Our results show that some high-pass filters preserved overall performance, and more importantly, enhanced performance in mammograms taken prior to development of visible, actionable abnormalities. This is the first time specific spatial frequencies bands have been identified in the radiological images that when enhanced improved very early cancer detection without impeding detection of obvious cancer lesions. These findings provide a promising avenue of using high-pass filtering image enhancements to improve gist of abnormality risk factors to be used as a low-cost individualized risk factor.

## Methods

### Participants

A total of 34 participants took part in this experiment, which was conducted in two versions, an in-person experiment and an online experiment. The online version was set up to avoid in-person contact during the COVID-19 pandemic. All participants were radiologists with experience reading mammograms in a clinical setting, which was defined as having read at least 1000 scans in the last year.

Sixteen participants took part in the in-person version of the experiment (9 female, 32 to 64 years old, mean 50.7+-10.8). They read on average 5056 scans (std 3707, range 1000 to 12000) over the last year, average 22 years in practice (std 11.6years, range 2 to 37), and on average spend 59% of their time diagnosing mammograms (std 34%, range 10 to 100%) in their work. Eighteen participants took part in the online version of the experiment (13 female, 33 to 67 years old, mean 46.9 +- 10.1). They read on average 5694 scans (std 2996, range 1000 to 10000) over the last year, average 14 years in practice (std 10.6 years, range 2 to 37), and on average spend 70% of their time diagnosing mammograms (std 27.1%, range 25 to 100%) in their work. The 5 radiologists at the lower end of cases read in the last year (<2000) had been practicing for 7, 18, 19, 30, and 37 years respectively, indicating extensive experience. Details for the demographics for each individual can be found in Table 1.

**Table 1:** Demographics for each participating radiologist. The table shows the age, gender, years of experience, percent of cases viewed that were mammograms, and number of cases viewed in the previous year.

Rad	Age	Gender	Years of Experience	Percent mammograms	Cases viewed	Group
**1**	32	F	2	100	4800	In person
**2**	44	F	12	50	6000	In person
**3**	63	M	35	15	2100	In person
**4**	44	F	10	100	3000	In person
**5**	42	F	14	100	4000	In person
**6**	62	M	34	25	12000	In person
**7**	60	F	29	100	6000	In person
**8**	63	M	38	80	10000	In person
**9**	35	F	11	80	12000	In person
**10**	63	F	36	100	2000	In person
**11**	63	M	37	25	1000	In person
**12**	56	F	30	20	1500	In person
**13**	38	F	8	70	9000	In person
**14**	56	M	24	50	5000	In person
**15**	44	M	18	10	1000	In person
**16**	46	M	19	25	1500	In person
**17**	65	M	42	100	8000	Online
**18**	48	F	10	75	6000	Online
**19**	35	F	7	80	10000	Online
**20**	43	M	12	25	1000	Online
**21**	62	F	16	100	10000	Online
**22**	38	F	7	50	1000	Online
**23**	67	M	37	30	3500	Online
**24**	43	M	11	25	2000	Online
**25**	43	F	14	50	5000	Online
**26**	37	F	6	50	8000	Online
**27**	45	F	11	95	9000	Online
**28**	48	F	9	75	9000	Online
**29**	35	F	7	80	8000	Online
**30**	46	F	3	100	2000	Online
**31**	49	F	12	100	5000	Online
**32**	48	F	-	100	7000	Online
**33**	33	M	4	40	3000	Online
**34**	60	F	24	80	5000	Online
**In person**	*50*.*69*	*9 F*	*22*.*31*	*59*.*38*	*5056*	
**Online**	*46*.*94*	*13 F*	*13*.*65*	*69*.*72*	*5694*	
**Overall**	*48*.*71*	*22 F*	*17*.*85*	*64*.*85*	*5394*	

Participants were recruited in-person during the Radiological Society of North America (RSNA) 2018 conference, and online over a period from 2020 to 2022, with recruitment emails sent to individual contacts, collaborating hospitals in the United Kingdom, and newsletters of various radiology profession groups in the UK and the Netherlands. The sample size of the radiologist groups was dictated by the availability of participants. This study was approved by the Psychology Departmental Ethics Committee of the University of York (ID 307), and all participants gave informed consent either written on paper (in-person) or digitally by clicking a button “I understand and agree” after reading the consent form (online).

### Stimuli and apparatus

The stimuli used in this experiment were de-identified bilateral mammograms sourced from the Complex Cognitive Processing Lab database of stimuli, in mediolateral oblique (MLO) or craniocaudal (CC) view. Four mammogram categories were used: normal mammograms of healthy women (normal), mammograms with obvious cancerous abnormalities (obvious), mammograms with subtle cancerous abnormalities (subtle), and mammograms without visibly actionable lesions taken three years prior to sign of abnormality (priors). Normal mammograms were defined as cases without abnormalities, of which the woman did not develop cancer in the next three years. Obvious and subtle mammograms were selected from a set of mammograms containing an abnormality, which were conspicuity-rated by an experienced mammogram-reading radiologist based on the visibility of the abnormality (obvious, subtle). Priors were defined as mammograms without any visible cancerous abnormalities of women who were then found to have developed cancer within the next three years retrospectively.

**Fig 1:**
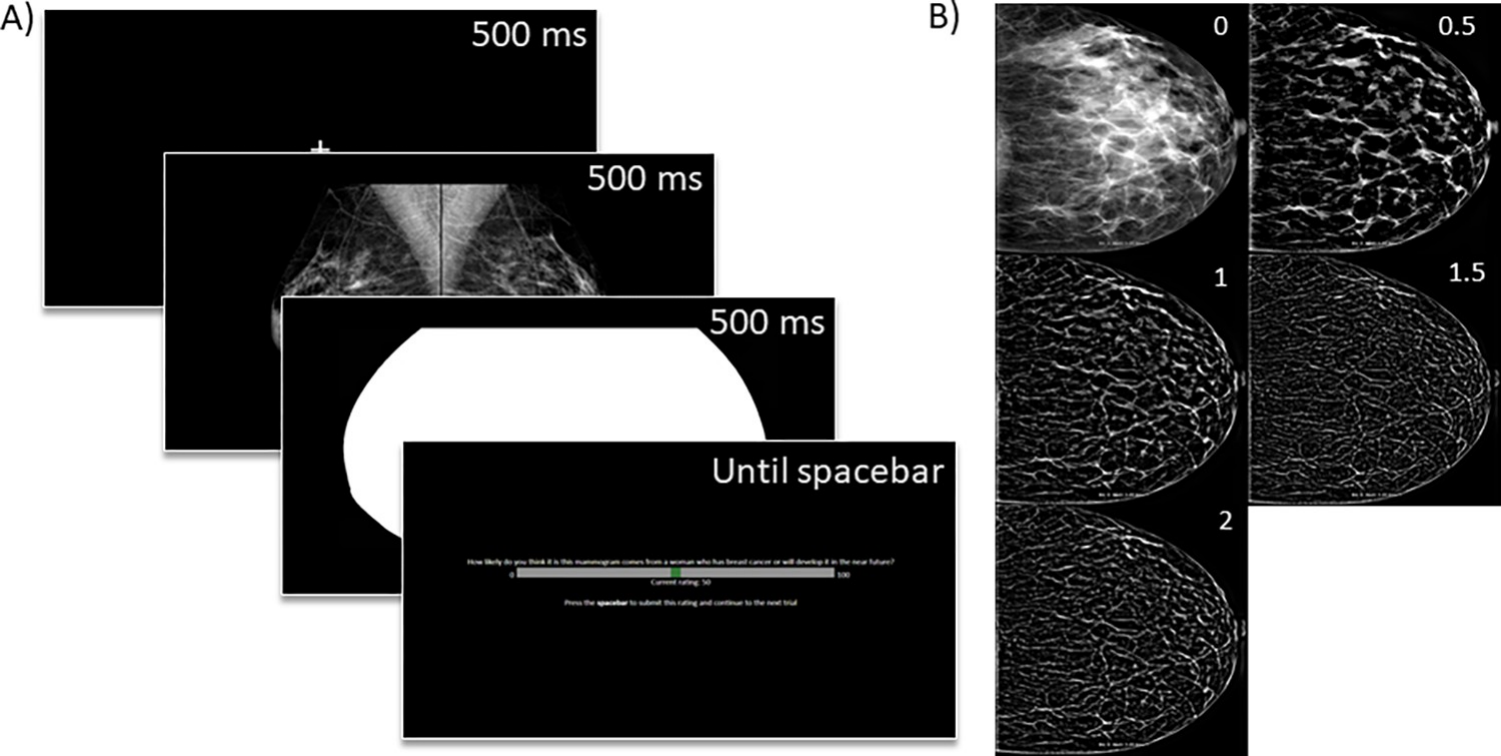
Procedure and stimuli used in the experiment. (A) Example visualization of the different screens in one trial, showing the fixation cross, mammogram case, mask, and rating screen. (B) Examples for the unaltered (0) and high-pass filtered versions (0.5, 1, 1.5, and 2 cpd) of a unilateral mammogram.

MATLAB was used to create the spatially filtered stimuli. Stimuli were filtered using a high-pass 2^nd^ order Butterworth filter with four different cut-off points. Filtered stimuli were brightened using a custom setting multiplying any pixel values above 10 (out of a 0 to 255 scale) by 3.5. Next, the filtered images were contrast normalized with the SHINE Toolbox for each group of filtered images together with the unfiltered images. Contrast normalization removes effects from overall differences in brightness between the filter groups. Four groups of spatially filtered images were created, namely 0.5, 1, 1.5, and 2 cycles per degree (cpd), examples of which can be seen in Fig 1B.

The in-person experiment was run using MATLAB, utilizing the Psychophysics Toolbox 3 extensions [23,24]. The online experiment was run on a custom web page. Participants were instructed to sit at a comfortable viewing distance of approximately 57 cm. In-person, stimuli were presented on a 17’ inch Dell colour display (1920 x 1200 pixels) with an 85 Hz refresh rate. For the online experiment, participants performed the experiment on their own laptop or PC. For the online experiment, a screen calibration method based on the work by Li, Joo [25] was used to ensure the stimuli were presented at 10 degrees of visual angle in height.

### Procedure

The experiment consisted of 3 practice trials followed by 3 blocks of test trials. In the practice trials, participants were familiarized with the display and rating screen, and were given feedback on the stimulus (normal or abnormal) after they confirmed their rating. In the test trials, no feedback was given. Each trial started with a fixation cross in the centre of the screen (500 ms), followed by the bilateral mammogram being shown for 500 ms. Then, a mask consisting of the solid white shape of the breast tissue was shown for 500 ms. Next, the rating screen appeared, on which moving the mouse changed the rating on a scale from 0 to a 100. Pressing the spacebar would confirm the current rating, after which the next trial automatically started (Fig 1A).

Participants were asked to rate how certain they were that the image came from a woman with breast cancer, or who will develop it in the near future. Participants were asked to adopt a liberal call back criterion, while being as accurate as possible. There was no time constraint for the rating in either condition, but participants were asked to report their first impression. During the in-person experiment, ratings were made on a scale from 0 (abnormal) to 100 (normal), while the online experiment used a scale from 0 (normal) to 100 (abnormal), due to a difference in coding. This is not expected to be any hindrance in comparing the two experiments, as the rating scale was clearly labelled in the instructions and on each rating screen, and 3 practice trials were available.

As previously stated, each participant completed three blocks of test trials. The same mammograms were used in each test block, to allow for direct comparison of performance. Each test block consisted of 120 trials: 60 normal, 20 obvious abnormal, 20 subtle abnormal, and 20 prior abnormal, in randomized order. One of the blocks always showed unaltered mammograms (F0) to ensure a baseline of performance, and the two other blocks showed two out of the four possible filter groups. Selected blocks and their order were randomized, although the switch from in-person to online measurements caused a lower number of participants for the F1 filter and the F1.5 filters than the F0.5 and the F2 filter. In total, all 34 participants rated F0, 21 participants rated F0.5, 15 participants rated F1, 13 participants rated F1.5, and 19 participants rated F2.

### Data analysis

To analyse our data, a signal detection theory framework was used to calculate performance measures, as previously described in an earlier publication [26]: “Given a rating, a mammogram was considered to be classified as either “abnormal” or “normal”, depending on whether the rating is higher or lower than some threshold. That classification was then compared to the ground truth. Signal detection measures were used to separately assess performance and response biases of the observer. Performance was represented by the D’ measure (D’ = z(true positive rate)–z(false positive rate)), where z denotes the inverse normal or z-transformation of the rates). In cognitive literature, d’ is referred to as “sensitivity”. However, “sensitivity” refers to the “true positive” or “hit” rate in the medical literature. We will refrain from using the term in order to avoid confusion. Response bias was measured by the criterion value, C (C = (z(true positive rate) + z(false positive rate))/-2). A negative criterion means that the observer was more likely to label the item as abnormal while a positive criterion means that observer was more likely to label the item as normal.

Receiver operating characteristic curves (ROC) were constructed by repeating this division of trials into proportions of true positive (hits) and false positive (false alarms) using different normal/abnormal rating cut-offs (here, 1 to 99). The area under the curve (AUC) of an ROC, ranging from 0.0 to 1.0, represents the probability that a randomly chosen abnormal case will be rated higher than a randomly chosen normal case [27]. Chance performance yields an AUC of 0.5. Higher AUCs indicate better performance in detecting the signal of cancerous abnormalities”.

Additionally, a technique for averaging ROCs from multi-reader, multi-case datasets was used to calculate an average ROC for visualization purposes [28]. D’ and criterion were derived using a rating cut-off of 50, as this is the middle point of the rating scale. AUCs were calculated across the entire rating scale and were calculated using the *sklearn*.*metrics auc* function in Python. These performance measures were calculated per participant for each of the filter conditions and mammogram category (obvious, subtle, and prior) combinations. Pre-processing into signal detection measures was performed in Python 3 using the following packages: json, scipy.stats, numpy, glob, sklearn.metrics auc, and csv. Next, statistical analysis was performed using SPSS 28.0.0.0 (190) for the univariate analysis of variance. For the primary analysis using linear mixed models, we used R version 4.1.3 in RStudio, and the following packages: tidyverse, lme4, sjPlot, rstatix, ggpubr, and emmeans. Additionally, boxplot figures were created using ggplot’s geom_boxplot function. These boxplots follow the standard arrangement, except for the whiskers, which contain 1.58 times the inter-quartile range, which is approximately equivalent to the 95% confidence interval of the data [29].

Firstly, univariate analysis of variance was performed to determine if there was any between-subjects difference in performance between the in-person and online groups of participants, using group as fixed factor, adding number of cases read as a covariate as previous research has shown a clear positive correlation between cases read and gist performance [19]. As no main effect of group was found, the two groups could be merged into one dataset.

The primary goal of this study was to investigate the effects of each high-pass filter on performance per image type relative to the unfiltered condition, for which a linear mixed model was used. The model was run separately for D’, criterion, and AUC, each with the factors Category (3 levels: Obvious, Subtle, Prior), and Frequency (5 levels: F0, F0.5, F1, F1.5, and F2), an Interaction factor between Category and Frequency, and a random intercept factor for participant ID to model individual differences. Akaike Information Criterion (AIC) [30] was used to estimate the goodness-of-fit including a penalty for the number of parameters included in the model, where a smaller AIC represents a better fit.

To investigate whether the category, frequency, and interaction factor contributed significantly to the fit of the mixed model, the full model was compared to a trimmed model in which one of these factors was removed. This was analysed using a log likelihood ratio test with the analysis of variance (ANOVA) function in R. If the full model was significantly better than the trimmed model, this provided evidence that this factor contributes significantly. For each factor that contributed significantly, post-hoc comparisons of the model estimates were used to investigate which specific levels of the factors differed from each other. These comparisons used Tukey corrections for multiple comparisons and Kenward-roger’s degrees-of-freedom method.

## Results

### Overall performance

**Fig 2:**
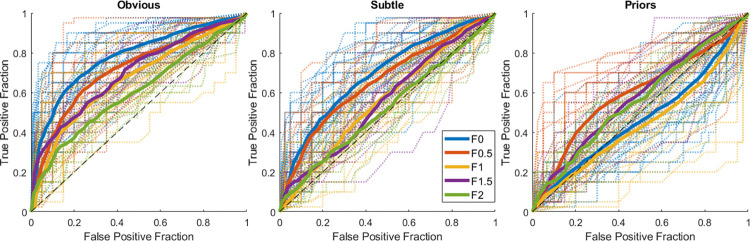
ROC curves per image category. Average and individual (dotted) ROCs per frequency condition (0, 0.5, 1, 1.5, and 2 cpd) for each abnormal mammogram category (obvious, subtle, and prior). The black dashed line represents chance levels, with anything above it being above chance.

Overall performance was above chance, replicating previous findings: Average D’ was above 0 and the AUC was above 0.5. Criterion values above 0 show that participants were biased towards conservative ratings. Estimated means from mixed models illustrate how these estimates follow the same patterns as the real data (Table 2). Performance was above chance for most participants across filter conditions for obvious and subtle abnormalities, shown by individual ROC curves above the chance line (Fig 2). However, for priors, performance was markedly lower or at chance for some participants in some filter conditions. Overall, participants could extract the gist of abnormality across all filter conditions but regularly struggled with prior caappeses, which will be further explored in the mixed models.

**Table 2:** Group average and mixed model estimated mean of of D’, criterion, and AUC for unfiltered mammograms and each high-pass filter frequency.

FREQUENCY	D’		AUC		CRITERION	
	Average	Estimated	Average	Estimated	Average	Estimated
**F0**	0.685	0.715	0.645	0.640	0.193	0.184
**F0.5**	0.937	0.897	0.657	0.665	0.469	0.27
**F1**	0.390	0.297	0.557	0.551	0.538	0.514
**F1.5**	0.666	0.708	0.611	0.617	0.790	0.879
**F2**	0.277	0.318	0.562	0.542	0.301	0.664

Univariate analysis of variance showed no significant effect of group (in-person/online) on D’ for unfiltered mammograms when accounting for number of cases read in the previous year (covariate) (corrected model F(2,31) = 2.198, p = .128). This supports the decision to combine the data from the two groups for the main analyses.

### Factors influencing D’ performance measure

For D’, linear mixed model analysis showed evidence for significant contributions of Category, Frequency, and an Interaction (intercept: 1.264, random effect of ID: 0.071, AIC: 406.62). An ANOVA comparing log-likelihoods of the full model to one without the category factor showed a significant contribution of category to the model fit (χ^2^(2) = 127.14, p = < .001). Similarly, frequency contributed significantly to the model fit (χ^2^(4) = 43.514, p = < .001), as did the interaction factor (χ^2^(8) = 51.655, p = < .001).

**Fig 3:**
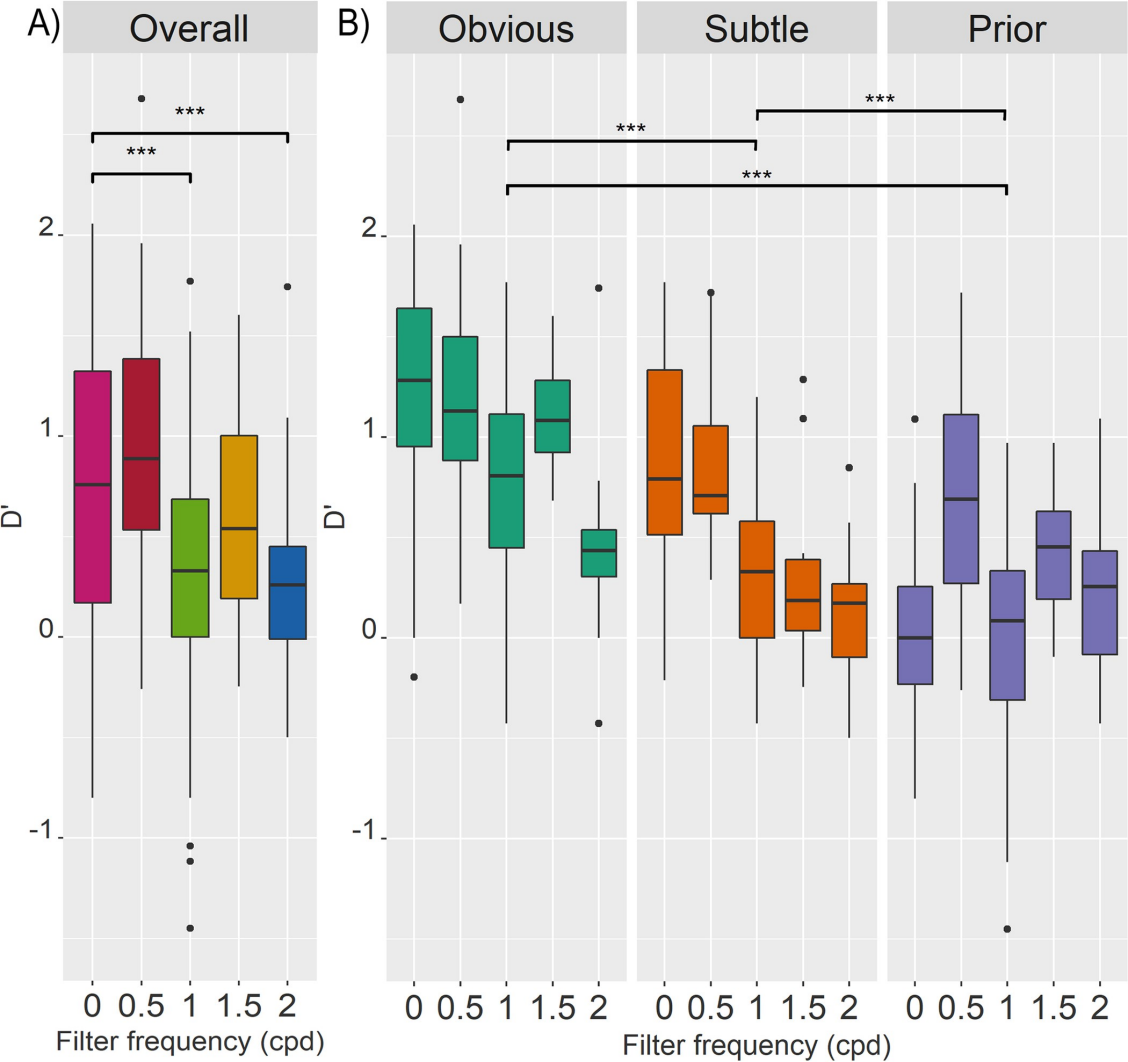
Boxplots of D’ across conditions. Each boxplot shows the median as the line within the coloured box containing the 25^th^ and 75^th^ percentiles, with extending whiskers containing the 95% CI, with any outliers plotted as dots. Significance of pairwise comparisons is indicated in the figure with * = p < .05, ** = p < .01,*** = p < .001. (A) Boxplots showing D’ across frequency conditions (0, 0.5, 1, 1.5, and 2 cpd). Pairwise comparisons of frequency showed that D’ was significantly higher for F0 than F1 (estimated difference = 0.482, t(271) = 6.080, p = < .0001) and F2 (estimated difference = 0.397, t(282) = 4.502, p = < .0001), but did not differ significantly from F0.5 (estimated difference = -0.181, t(273) = -2.637, p = .067), and F1.5 (estimated difference = 0.007, t(282) = 0.083, p = 1.000)–and even trended towards a higher D’ in F0.5. (B) Boxplots showing D’ for each mammogram category (obvious, subtle, and prior) and frequency, to illustrate mammogram category and interaction effects. Pairwise comparisons of mammogram category showed that D’ was significantly higher for obvious than subtle (estimated difference = 0.462, t(258) = 7.278, p = < .0001) and prior mammograms (estimated difference = 0.683, t(258) = 10.763, p = < .0001), and higher for subtle than prior mammograms (estimated difference = 0.221, t(258) = 3.485, p = < .001).

Pair-wise comparisons were performed for frequency (Fig 3A), and mammogram category (Fig 3B). Based on these comparisons, specific interaction effects were reviewed, comparing the unaltered mammograms to the 0.5 and 1.5 cpd high-pass filters that showed no significant difference in overall D’. For priors, D’ was significantly higher for F0.5 (estimated difference = 0.646, t(263) = 5.566, p = < .001) and F1.5 (estimated difference = 0.499, t(268) = 3.443, p = .006) than unfiltered (F0) mammograms. Meanwhile, there was no significant difference in D’ between F0 and F0.5 for obvious (estimated difference = 0.091, t(264) = 0.781, p = .936) or subtle (estimated difference = 0.011, t(263) = 0.098, p = 1.000) mammograms. For F0 versus F1.5, there was no difference in D’ for obvious mammograms (estimated difference = 0.068, t(268) = 10.467, p = .990), but F1.5 reduced D’ for subtle mammograms (estimated difference = 0.453, t(268) = 3.128, p = .017).

#### Factors influencing AUC performance measure

The same pattern of results was observed for AUC. For AUC, the linear mixed model analysis showed evidence of significant contributions of Category, Frequency, and an Interaction factor. The full model had an intercept of 0.760, and a random effect of ID intercept of 0.002, and an AIC of -589.77. An ANOVA comparing the log-likelihoods of the full model to the model without the category factor showed a significant difference (χ^2^(2) = 168.97, p = < .001), showing that category significantly adds to the model fit. Similarly, the frequency factor contributes significantly compared to a model without this factor (χ^2^(4) = 46.627, p = < .001). Lastly, the interaction factor was also significant (χ^2^(8) = 75.396, p = < .001).

**Fig 4:**
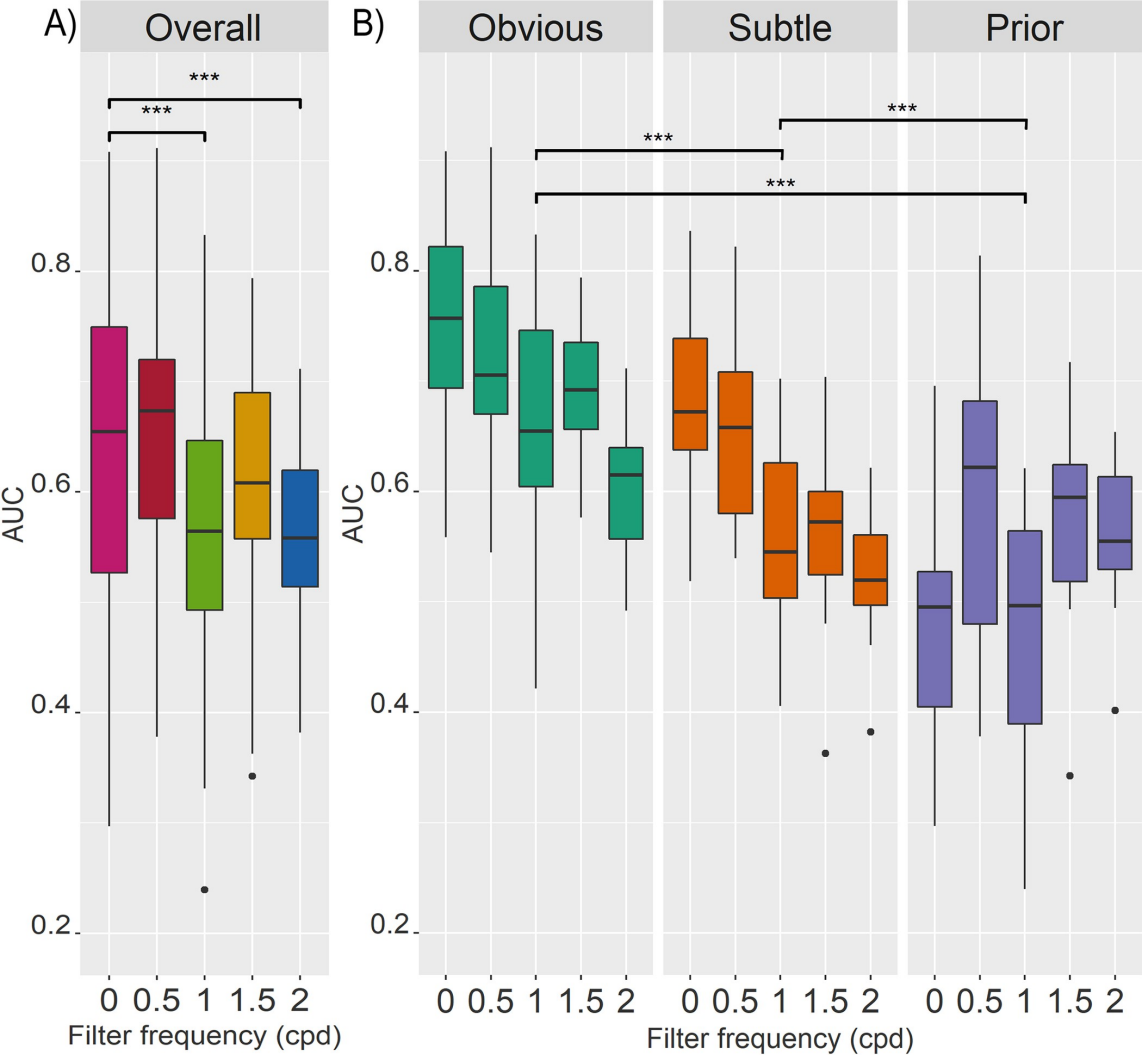
Boxplots of AUC across conditions. Each boxplot shows the median as the line within the coloured box containing the 25^th^ and 75^th^ percentiles, with extending whiskers containing the 95% CI, with any outliers plotted as dots. Significance of pairwise comparisons is indicated in the figure with * = p < .05, ** = p < .01,*** = p < .001. (A) Boxplots showing AUC across frequency conditions (0, 0.5, 1, 1.5, and 2 cpd). Pairwise comparisons of frequency showed that AUC was significantly higher for F0 than F1 (estimated difference = 0.089, t(273) = 6.533, p = < .0001) and F2 (estimated difference = 0.098, t(284) = 5.647, p = < .0001), but did not differ significantly from F0.5 (estimated difference = -0.025, t(273) = -1.827, p = .360) and F1.5 (estimated difference = 0.023, t(284) = 1.306, p = .688). (B) Boxplots showing AUC for each mammogram category (obvious, subtle, and prior) and frequency, to illustrate mammogram category and interaction effects. Pairwise comparisons of mammogram category showed that D’ was significantly higher obvious than subtle (estimated difference = 0.089, t(258) = 7.058, p = < .0001) and prior mammograms (estimated difference = 0.156, t(258) = 12.368, p = < .0001), and higher for subtle than prior mammograms (estimated difference = 0.068, t(258) = 5.310, p = < .001).

Pair-wise comparisons were performed for the frequency (Fig 4A), as well as mammogram category factors (Fig 4B). Again, interaction effects were reviewed with a special focus on the F0.5 and F1.5 groups that showed no significant difference in overall AUC compared to F0. These comparisons showed that AUC for prior mammograms was significantly higher for F0.5 (estimated difference = 0.134, t(264) = 5.844, p = < .001) and F1.5 (estimated difference = 0.110, t(270) = 3.843, p = < .001) than the unfiltered F0 group. Meanwhile, there was no significant difference in AUC between F0 and F0.5 for obvious (estimated difference = 0.036, t(264) = 1.568, p = .519) or subtle (estimated difference = 0.024, t(264) = 1.033, p = .840) mammograms. On the other hand, for F0 versus F1.5, there was no difference in AUC for obvious mammograms (estimated difference = 0.055, t(270) = 1.931, p = .303), but there was a reduction in AUC for subtle mammograms at F1.5 (estimated difference = 0.122, t(270) = 4.292, p = < .001). These interactions can also be observed in Fig 4B.

#### Factors influencing the bias in rating measure

**Fig 5:**
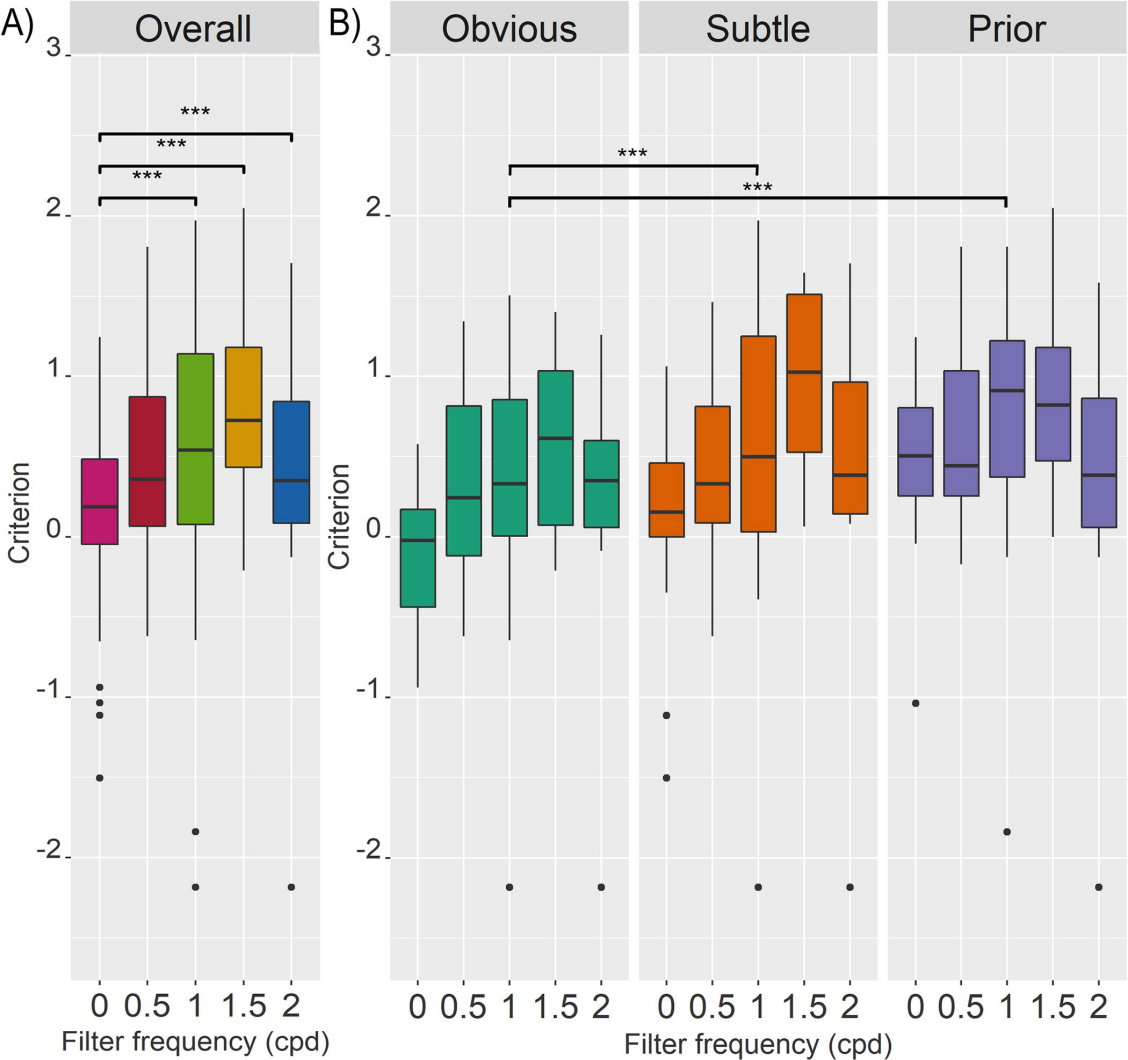
Boxplots of criterion across conditions. Each boxplot shows the median as the line within the coloured box containing the 25^th^ and 75^th^ percentiles, with extending whiskers containing the 95% CI, with any outliers plotted as dots. Significance of pairwise comparisons of main effects is indicated in the figure with * = p < .05, ** = p < .01,*** = p < .001. (A) Criterion across frequency conditions (0, 0.5, 1, 1.5, and 2 cpd). Pairwise comparisons of frequency showed that criterion was significantly higher for F0 than F1 (estimated difference = -0.3295, t(261) = -5.640, p = < .0001), F1.5 (estimated difference = -0.695, t(264) = -9.176, p < .0001), and F2 (estimated difference = -0.480, t(264) = -6.329, p = < .0001), but did not differ significantly from F0.5 (estimated difference = -0.086, t(261) = -1.477, p = .579). (B) Criterion for each mammogram category (obvious, subtle, and prior) and frequency, to illustrate mammogram category and interaction effects. Pairwise comparisons of mammogram category showed that criterion was significantly lower (less conservative) for obvious than subtle (estimated difference = -0.232, t(258) = -4.345, p = < .0001) and prior mammograms (estimated difference = -.347, t(258) = -6.517, p = < .0001), but did not differ significantly between subtle and prior mammograms (estimated difference = -.116, t(258) = -2.172, p = .078).

For criterion, linear mixed model analysis showed evidence of significant contributions of Category, Frequency, and an Interaction (intercept: -0.108, random effect of ID: 0.323, AIC: 356.35). An ANOVA comparing log-likelihoods of the full model to one without category showed a significant contribution of category to model fit (χ^2^(2) = 48.458, p = < .001). Similarly, frequency (χ^2^(4) = 53.488, p = < .001) and the interaction effect (χ^2^(8) = 16.563, p = .035) contributed significantly to the model fit. Pairwise comparisons of main effects can be observed in Fig 5, showing that participants became more conservative for all filter conditions except F0.5.

## Discussion

D’ and AUC mixed model findings demonstrate that F0.5 and F1.5 high-pass filters significantly increased gist extraction performance in mammograms acquired years prior to onset on any visible cancerous lesions: D’ was boosted by 0.646 for F0.5 and by 0.499 for F1.5 respectively, a considerable increase. Additionally, 0.5 cpd high-pass filters did not impact radiologists’ performance on obvious or subtle mammograms. This strongly suggests that removing the lowest frequencies in mammograms can enhance the gist of abnormality for current presence or future risk of cancer in cases that do not yet show any visibly actionable signs of cancer, while retaining the signal of current abnormalities.

Radiologists rated mammograms that maintained only frequencies over 1, 1.5 and 2 cpd more conservatively compared to those with frequencies above 0.5 cpd or those with full spectrum. Thus, filtering out spatial frequencies below 0.5 cpd would be the most suitable, as it did not significantly affect observer’s decision criterion, retained performance for obvious and subtle mammograms, and enhanced it for priors. Gist ratings for these high-pass filtered mammograms could be used to flag missed current cancers for a second opinion and for enhanced screening when no abnormalities are found.

Out of the tested filter conditions, two (F0.5 and F1.5) showed retained overall performance and increased performance on priors. However, the other two filter conditions (F1 and F2) showed an overall drop in performance without increasing performance for any sub-types. This pattern could be explained by different effects influencing performance. Firstly, frequencies below 0.5 cpd might mask gist signals, especially in priors, resulting in an increase in performance when a F0.5 filter is applied, perhaps because this removes widespread ‘blur” from breast density. While breast density can be a risk factor for breast cancer, previous research found no correlation between BIRAD density and gist of abnormality ratings [17–19,31]. Secondly, intermediate frequencies between F0.5 and F1 might include some important aspects of the gist signal, causing a significant drop in performance when filtering below F1. Thirdly, increased performance on priors with a slight decrease for subtle abnormalities when removing signal between F1 and F1.5 suggests that this frequency band contain some gist signal, but also contributes noise that might obscure global signals of (future) cancer. Lastly, reduced performance when spatial frequencies below 2 cpd are removed from mammograms points to the importance of F1.5 –F2 cpd for the gist signal. Together, these findings suggest the gist of abnormality is contained mainly in 0.5 to 1 cpd and 1.5 to 2 cpd spatial frequencies, with a mix of signal and noise in 1 to 1.5 cpd. Further research would be needed to test these predictions in detail.

The combined effect of high-pass filtering and contrast normalization in increasing the performance of radiologists matches previous findings in both behavioural and neuroimaging work on spatial frequency. Our results match the previous observation that low-pass filtering strongly reduced gist of abnormality performance, while high-pass filtering without contrast normalization had a much less pronounced effect [18]. Similarly, in scenes gist performance on HSF scenes was reduced without contrast normalization, but contrast normalization equalized performance between LSF and HSF scene images [22]. Our findings match this retention of overall performance with HSF with contrast normalization, combined with a novel enhancement of global abnormality signals in priors.

What is more, recent neuroimaging work shows that many scene-selective areas respond preferentially to HSF rather than LSF. Activity in the parahippocampal place area (PPA) was higher for HSF than LSF checkerboards, scenes, and faces [32]. Similarly, contrast-equalized HSF scenes activated the PPA and the occipital place area (OPA) more than LSF equivalents, although there was no difference in the retrosplenial cortex (RSC) [33]. Going beyond simple levels of activation, computational models can decode scene categories from BOLD signals in the PPA, RSC, and lateral occipital complex (LOC) of viewing photographs and line drawings (= HSF) [34]. Similarly, scene category could be decoded from HSF photographs viewed for 800 ms in the PPA, RSC, LOC, and OPA, while LSF photographs could only be decoded in the posterior PPA [35]. This increased activation and decoding in response to HSF demonstrate the important role of HSF’s contours and edges in rapid scene category processing. This fits with our behavioural findings of importance for HSF for mammogram-category extraction. There might be a similar role for HSF in both scene and medical abnormality gist extraction, again strengthening our belief that mammogram perception closely resembles scene perception.

Our filtering protocol included a brightness increase and contrast normalization. This method made the fine detail more visible in the filtered mammograms. A minor disadvantage is that this makes the data less informative for understanding the role of high spatial frequencies in conventional mammograms, as boosted brightness increased the weight given to the high frequency information. However, these stimuli remain ecologically valid, as no mammogram is ‘unaltered’. X-ray methodology creates a 2D representation of 3D tissue density, and the visibility of specific tissues depends on the specific machine, settings, image processing used [36], and even the practitioners’ preferential compression force [37]. What’s more, programs used for viewing medical cases often contain options to change the contrast or brightness. Thus, a brightness increase would not make the mammogram more or less ‘naturalistic’, it simply increased the chance of finding high-pass filters that enhanced detection rates, which was the main objective of this study.

Future research could focus on more fine-tuned enhancements by delving into the role of specific spatial frequency bands using bandpass or bandstop filters, which combined low- and high-pass filters to selectively retain *or* filter out a small band of frequencies. This would allow for more controlled adjustment of frequency content and could help identify the exact combination of spatial frequencies that contain the gist of abnormality. This could for example be used to filter out F0 –F0.5 and F1-F1.5 to investigate whether this combination further enhances the gist signal.

It might also be worth considering whether these, or similar image enhancements have the same effects on different domains of medical imaging. Previous research has shown that a gist of abnormality signal is also detectable in various other imaging modalities, such as digital breast tomosynthesis [38], chest radiographs [16,39], and even pap test images (micrographs) of cervical cells [17]. It is possible that a similar high-pass filter would increase the signals of abnormality in other medical images as well, especially for radiographs, but it is also conceivable that different tissues are differentially affected by the development of a cancerous abnormality and would require different spatial frequency filtering to enhance their gist of abnormality signals. By comparing effects on different imaging modalities future studies could investigate the best image enhancements for each, which could in addition give insight into the (dis)similarities in gist signal content between modalities.

## Conclusion

In conclusion, we have shown that certain high-pass filters (F0.5 and F1.5 cpd) combined with brightness boosting and contrast normalization can retain overall performance while boosting the gist of abnormality signal in mammograms at future cancer risk. Especially the 0.5 cpd high-pass filter seemed promising in boosting the signal in priors, without reducing the signal in mammograms with obvious or subtle signs of cancer in mammograms, nor making the radiologists more conservative in their decisions.

The current study clearly identifies specific spatial frequency bands that when enhanced improved very early cancer detection without impeding detection of obvious cancer lesions, creating a way to improve patient outcomes and a way for low-cost individualized medicine. Our findings are based on a sizeable sample of 34 radiologists across a range of countries and clinical practices, making them more generalizable than previous more homogenous samples. Our findings have clear clinical importance and implementation feasibility because while enhancing certain spatial frequencies bands increased performance it did not change the decision criteria of the radiologists.

Future research could investigate the effects of image enhancements on additional medical imaging modalities, to explore whether these findings hold true across imaging types. Additionally, future experiments should use bandpass or bandstop filtering to selectively retain or remove spatial frequencies to further investigate the role of specific spatial frequency bands in mammograms. The approach could be used to inform about more subtle enhancements that could potentially further boost the gist signal allowing for even earlier cancer detection. Overall, our findings provide initial evidence for a viable solution to enhance the gist of abnormality in mammograms to use as a risk factor in the clinical toolbox for radiologists.

## Abbreviations

Cpd: cycles per degree
ROC: receiver operator curve
AUC: area under the curve
AIC: Akaike Information Criterion
LSF: Low spatial frequencies
HSF: high spatial frequencies
